# Sulforaphane attenuates pulmonary fibrosis by inhibiting the epithelial-mesenchymal transition

**DOI:** 10.1186/s40360-018-0204-7

**Published:** 2018-04-02

**Authors:** Sun Young Kyung, Dae Young Kim, Jin Young Yoon, Eun Suk Son, Yu Jin Kim, Jeong Woong Park, Sung Hwan Jeong

**Affiliations:** 1 Department of Internal Medicine, Gachon University Gil Medical Center, 21 Namdong-daero 774, Namdong-gu, Incheon, 21565 Republic of Korea; 2 Department of Biological Science, College of Bio-nano Technology, Gachon University, Seongnam-daero 1342, Seongnam, South Korea

**Keywords:** Idiopathic pulmonary fibrosis, Bleomycin, Sulforaphane, Epithelial-mesenchymal transition

## Abstract

**Background:**

Idiopathic pulmonary fibrosis (IPF) is a progressive and fatal disease with no effective treatment. The epithelial-mesenchymal transition (EMT) is a critical stage during the development of fibrosis. To assess the effect of sulforaphane (SFN) on the EMT and fibrosis using an in vitro transforming growth factor (TGF)-β1-induced model and an in vivo bleomycin (BLM)-induced model.

**Methods:**

In vitro studies, cell viability, and cytotoxicity were measured using a Cell Counting Kit-8. The functional TGF-β1-induced EMT and fibrosis were assessed using western blotting and a quantitative real-time polymerase chain reaction. The lungs were analyzed histopathologically in vivo using hematoxylin and eosin and *Masson’s trichrome* staining. The BLM-induced fibrosis was characterized by western blotting and immunohistochemical analyses for fibronectin, TGF-β1, E-cadherin (E-cad), and α-smooth muscle actin (SMA) in lung tissues.

**Results:**

SFN reversed mesenchymal-like changes induced by TGF-β1 and restored cells to their epithelial-like morphology. The results confirmed that the expression of the epithelial marker, E-cadherin, increased after SFN treatment, while expression of the mesenchymal markers, N-cadherin, vimentin, and α-SMA decreased in A549 cells after SFN treatment. In addition, SFN inhibited TGF-β1-induced mRNA expression of the EMT-related transcription factors, Slug, Snail, and Twist. The SFN treatment attenuated TGF-β1-induced expression of fibrosis-related proteins, such as fibronection, collagen I, collagen IV, and α-SMA in MRC-5 cells. Furthermore, SFN reduced the TGF-β1-induced phosphorylation of SMAD2/3 protein in A549 cells and MRC-5 cells. BLM induced fibrosis in mouse lungs that was also attenuated by SFN treatment, and SFN treatment decreased BLM-induced fibronectin expression, TGF-β1 expression, and the levels of collagen I in the lungs of mice.

**Conclusions:**

SFN showed a significant anti-fibrotic effect in TGF-β-treated cell lines and BLM-induced fibrosis in mice. These findings showed that SFN has anti-fibrotic activity that may be considered in the treatment of IPF.

**Electronic supplementary material:**

The online version of this article (10.1186/s40360-018-0204-7) contains supplementary material, which is available to authorized users.

## Background

Idiopathic pulmonary fibrosis (IPF) is a chronic, progressive, fibrotic lung disease characterised by expansion of fibroblast/myofibroblast populations and aberrant remodelling, which can lead to respiratory failure and death [1]. The major pathological findings of IPF are the expressional upregulation of connective tissue growth factor and transforming growth factor (TGF)-β1, fibroblast migration and proliferation, and extracellular matrix deposition [2, 3]. In IPF, the ability of alveolar epithelial cells to repair against recurrent microinjury is impaired and they secrete fibrogenic growth factors, such as TGF-β, and exhibit fibroblast/myofibroblast proliferation and activation. Furthermore, myofibroblast activation induces excessive accumulation of extracellular matrix components, which destroy the alveolar structure. Resident mesenchymal cell proliferation, epithelial mesenchymal transition (EMT), and circulating fibroblasts are likely sources of myofibroblasts [4].

EMT is a process whereby epithelial cells transition into cells of the mesenchymal phenotype, such as fibroblasts or myofibroblasts [5–7]. Recently, it has been recognised that EMT has important roles in embryogenesis, cancer progression, and organ fibrosis [5]. During fibrogenesis of several organs, EMT may be a major provider of pathogenic mesenchymal cell types, such as myofibroblasts [7]. EMT can be induced by various factors. For example, a wealth of evidence indicates that TGF-β is a major inducer of EMT [6, 8]. Growth factors downregulate genes expressed in epithelial cells, such as E-cadherin (E-cad), and upregulate genes normally expressed in mesenchymal cells, such as N-cadherin (N-cad), vimentin, and α-smooth muscle actin (α-SMA) [8, 9]. At the molecular level, EMT is characterised by downregulation of E-cad and cytokeratins. This process is controlled by a group of transcription factors referred to as EMT regulators, which include Snail, Slug, Twist, ZEB1, SIP1, and E12/47 [7, 8, 10, 11].

Although many immunomodulatory and anti-inflammatory drugs have been used to treat IPF, they do not prevent its progression [11, 12]. Recently, pirfenidone and nintedanib were found to be partially effective against IPF, and were approved by the Food and Drug Administration for mild-to-moderate IPF [1, 13, 14]. Unlike nintedanib, which is an inhibitor of multiple tyrosine kinases, pirfenidone has anti-inflammatory and antifibrotic effects, although no specific molecular target has been identified [15]. However, additional treatment trials are needed, because current treatments for IPF have limited efficacy.

Sulphoraphane (SFN) is a phytochemical that is mainly found in cruciferous vegetables, such as broccoli, cabbage, and Brussels sprouts, and its antioxidative effects are known to involve nuclear factor, erythroid-derived 2-related factor 2 (Nrf2)-mediated induction of phase II detoxifying enzymes [16, 17]. The chemopreventative effects of SFN are known to involve the induction of cell cycle arrest and apoptosis [18, 19]. Furthermore, recent studies have shown that it modulates various signalling pathways associated with oncogenic EMT [20, 21]. Several studies have reported that SFN has anti-fibrotic activity in hepatic fibrosis and airway smooth muscles [22, 23]. Nrf2 activation by SFN attenuates TGF-β signalling in hepatic fibrosis, and SFN treatment was found to induce Nrf2 expression and myofibroblastic dedifferentiation in IPF fibroblasts [22, 24]. Moreover, SFN was recently reported to prevent bleomycin-induced pulmonary fibrosis by inhibiting oxidative stress [25].

We hypothesised that SFN might show an anti-fibrotic efficacy in pulmonary fibrosis by inhibiting EMT. We assessed the effects of SFN on TGF-β1-induced EMT and fibrosis in a lung alveolar epithelial (A549) cell line and a fibroblast (MRC-5) cell line. In addition, we assessed the effects of SFN on a BLM-induced pulmonary fibrosis model in C57BL/6 mice followed by treatment with SFN for 4 weeks.

## Methods

### In vitro cell culture and sample treatment

The human type II alveolar epithelial A549 cell line (ATCC^®^ CCL-185^™^) and the human fibroblast MRC-5 cell line (ATCC^®^ CCL-171^™^) were purchased from the American Type Culture Collection (Manassas, VA, USA). The A549 cells were cultured in RPMI medium (Welgene, Seoul, Republic of Korea) supplemented with 100 U/mL penicillin, 100 μg/mL streptomycin, and 10% foetal bovine serum (Gibco, Grand Island, NY, USA). The MRC-5 cells were cultured in Dulbecco’s Modified Eagle’s Medium (Welgene) supplemented with 100 U/mL penicillin, 100 μg/mL streptomycin, and 10% foetal bovine serum (Gibco). The cells were treated with various concentrations of SFN (10 and 20 μM) for the indicated times. Cells were also treated with 0.1% ultrapure water as a vehicle control.

### In vivo BLM-induced pulmonary fibrosis in mice

Male C57BL/6 mice (3 weeks of age; body weight, 20–25 g) were purchased from Dae Han Biolink (Umsung, Republic of Korea). This animal study was approved by the Panel on Laboratory Animal Care of Gachon University (GIACUCR-011). The mice were fed a commercial diet (Cargill Agri Purina, Sungnam, Republic of Korea) together with tap water ad libitum. They were housed in an animal facility maintained at 20 ± 2 °C with 40 ± 10% humidity under a 12/12 h light/dark cycle. The mice were cared for according to the Guidelines of the Korean Food and Drug Administration and the United States National Institutes of Health Guidelines for the Care and Use of Laboratory Animals. BLM was purchased from Sigma-Aldrich (St. Louis, MO, USA). Twenty male mice were divided randomly into three experimental groups and a control group, with five mice per group, as follows: (1) treatment with distilled water (DW) (control), (2) treatment with BLM (5 units, twice) (BLM group), and (3) treatment with BLM and SFN (50 μg/kg) (BLM + SFN group). The control mice received only DW on days 0 and 14. BLM (5 units) was instilled intratracheally in a DW suspension on days 0 and 14. Intratracheal administration was performed using the “tongue-pull” method. SFN (50 μg/kg body weight) was orally administered three times a week for 28 days. At the end of the experiments, mice were sacrificed by euthanasia using isoflurane inhalation and lung tissue was collected.

### Histopathological analyses

The right lungs were embedded in paraffin wax, fixed in 10% formalin, and processed into sections. The sections were stained with haematoxylin and eosin (H&E) or subjected to Masson’s trichrome (M-T) staining and immunohistochemical (IHC) staining. The histopathological evaluation of pulmonary fibrosis was scored according to the density of M-T staining (the M-T score) and IHC staining using an image J analysis program.

### Hydroxyproline assay

To assess collagen accumulation, the hydroxyproline content in lung tissues was measured according to the protocol provided with a hydroxyproline test kit (BioVision, Milpitas, CA, USA). 10 mg of tissues were homogenized in 100 μl of DW. 100 μl of 12 M HCl were added to 100 μl of sample and hydrolyzed for overnight at 120 °C. The samples were centrifuged at 14,000 rpm for 20 min, and 10 μl of supernatant was transferred to a 96-well plate. 100 μl of chloramine-T reagent was added to the samples and incubated for 5 min at room temperature. After 100 μl of dimethylaminobenzaldehyde reagent was added to each sample and incubated for 90 min at 60 °C. The absorbance of the samples was measured using a microplate reader (Thermo LabSystems, Helsinki, Finland) at 560 nm.

### Cell viability assay

This assay method was based on the ability of a mitochondrial dehydrogenase enzyme from viable cells to reduce 3-(4,5-dimethylthiazol-2-yl)-2,5-diphenyltetrazolium bromide (MTT) into a dark blue formazan crystal that accumulated in cells. The A549 and MRC-5 cells were seeded at a density of 5 × 10^3^ cells/well in a 96-well plate and incubated at 37 °C for 24 h under a humidified 5% CO_2_ atmosphere. The cells were treated with various concentrations of SFN (10, 20, and 40 μM) for the indicated times with or without TGF-β1. At the end of the incubation, 10 μL of MTT solution (5 mg/mL in phosphate-buffered saline) was added to each well. After additional incubation at 37 °C for 4 h, the medium was gently removed and 100 μL of dimethylsulphoxide was added. The absorbance of samples was then measured using a microplate reader (Thermo LabSystems, Helsinki, Finland) at 550 nm.

### Western blot analyses

The protein was extracted from MRC-5 cells, A549 cells, and mouse lung tissue using a radioimmunoprecipitation assay buffer according to the manufacturer’s protocol. Then, 20–40 μg protein from each sample was separated by 10% sodium dodecyl sulphate-polyacrylamide gel electrophoresis and transferred to a polyvinylidene difluoride membrane (Millipore, Bedford, MA, USA). The membrane was blocked with 5% (*w*/*v*) non-fat skim milk for 60 min at room temperature and then incubated with the following primary antibodies: anti-p-SMAD2/3, anti-SMAD2/3, anti-TGF-β1, anti-E-cad, anti-N-cadherin, or anti-vimentin (Cell Signaling Technology, Danvers, MA, USA); anti-fibronectin or anti-type 1 collagen (Santa Cruz Biotechnology, Santa Cruz, CA, USA); or anti-α-SMA or anti-type 1 collagen (Abcam, Cambridge, UK) overnight at 4 °C. The membrane was then incubated with a 1:5000 dilution of horseradish peroxidase-conjugated secondary antibody for 1 h at room temperature. Each protein was detected using a chemiluminescence detection system according to the manufacturer’s protocol (Amersham ECL, Little Chalfont, UK). The band intensity was quantified by densitometric analyses using ImageJ software (National Institutes of Health, Bethesda, MD, USA).

### RNA extraction and quantitative real-time-polymerase chain reaction (qRT-PCR)

Expression levels of fibrosis-related genes and EMT related-transcription factors were determined by qRT-PCR. Total RNA was extracted using the RNAiso Plus reagent (Takara Bio, Dalian, China) according to the manufacturer’s instructions. The concentrations of all RNA samples were determined spectrophotometrically. The cDNA was produced from the total RNA (1 μg) using a Prime Script RT reagent kit (Takara Bio) according to the manufacturer’s protocol. The qRT-PCR was performed on a Bio-Rad iQ5 RT-PCR detection system (Bio-Rad, Hercules, CA, USA) using a SYBR Premix Ex Taq II kit (Takara Bio). All samples were run in triplicate, and glyceraldehyde 3-phosphate dehydrogenase (GAPDH) was used as an internal control. The expression levels were calculated from the PCR profiles of each sample using the threshold cycle (Ct), corresponding to the cycle with a statistically significant increase in fluorescence. To correct for differences in the amount of total cDNA in the starting reaction, the Ct values for the endogenous control (GAPDH) were subtracted from those of the corresponding sample.

### Statistical analysis

Values were expressed as means ± standard deviation (SD). A one-way analysis of variance was used to identify differences among groups. Tukey’s test was used to determine specific mean differences. All statistical analyses were performed using GraphPad Prism software (ver. 4.0; GraphPad, La Jolla, CA, USA). A value of *p* < 0.05 was considered to be significant.

## Results

### The effects of SFN on the TGF-β1-induced EMT in alveolar epithelial A549 cells

**Fig. 1:**
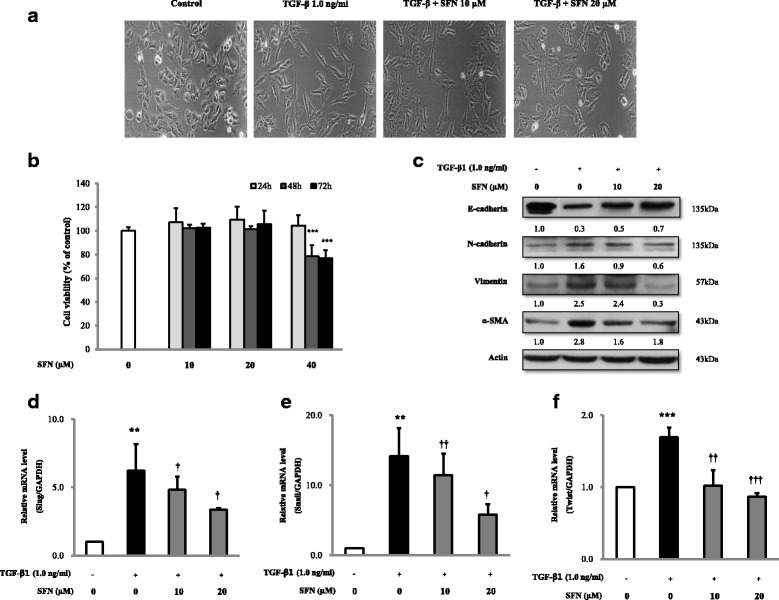
The effect of sulphoraphane (SFN) treatment on the morphology and protein markers in the transforming growth factor (TGF)-β1-induced epithelial-mesenchymal transition (EMT) in A549 epithelial cells. The cells were pre-treated with the indicated concentrations of SFN for 1 h and then stimulated with TGF-β1 (1.0 ng/ml) for 24 h except α-smooth muscle actin for 72 h. The effects of SFN on cell viability for 24, 48, and 72 h (**b**). SFN treatment restored the TGF-β1-induced changes in epithelial morphology with original magnification, × 200 (**a**). Western blot analysis of the epithelial cell marker (E-cadherin), and the mesenchymal markers (N-cadherin, vimentin, and α-smooth muscle actin) (**c**). The mRNA levels of EMT-related transcription factors including Slug (**d**), Snail (**e**), and Twist (**f**) were measured by the quantitative real-time polymerase chain reaction (qRT-PCR). The data are expressed as means ± standard deviation of at least three different experiments. ^*^p < 0.05, ^**^p < 0.01, ^***^p < 0.001 versus the control; ^†^*p* < 0.05, ^††^*p* < 0.01, ^†††^*p* < 0.001 versus TGF-β1 induction

Cell viability of A549 cells did not significant decrease at the concentration of 10 or 20 μM SFN (Fig. 1b). TGF-β1 treatment of A549 lung epithelial-like cells resulted in a spindle-like mesenchymal phenotype and the loss of cell-cell contact. Treatment with SFN caused reversion to the mesenchymal-like changes induced by TGF-β1 and restored the cells to their epithelial-like morphology (Fig. 1a). To investigate whether SFN could influence EMT-related protein expression, western blot analyses of A549 cell lysates showed that the expression of the epithelial marker, E-cad, increased after SFN treatment, while expression of the mesenchymal markers, N-cad, vimentin, and a-SMA, decreased in A549 cells (Fig. 1c). The effects of SFN treatment on EMT-related transcription factors were also analysed using RT-PCR. SFN treatment inhibited TGF-β1-induced mRNA expression of the EMT-related transcription factors, Slug, Snail, and Twist (Fig. 1d, e, f). These results suggested that transcription factors were involved in the TGF-β1-induced EMT inhibition after SFN treatment.

### The effects of SFN on TGF-β1-induced fibrosis in fibroblast MRC-5 cells

**Fig. 2:**
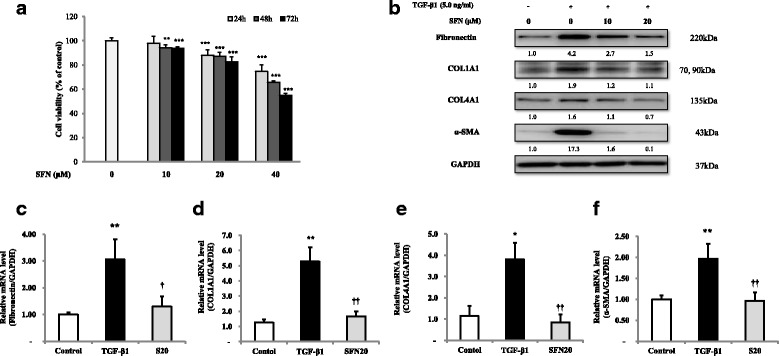
The effect of SFN on the expression of protein markers in TGF-β1-induced fibrosis in MRC-5 fibroblast cells. The cells were pre-treated for 1 h with the indicated concentrations of SFN and for 48 h with TGF-β1 (5.0 ng/ml). The effects of SFN on cell viability for 24, 48, and 72 h (**a**). The protein levels of fibronectin, collagen, and α- SMA were measured by western blot analyses (**b**). The mRNA levels of fibronectin (**c**), type I collagen (**d**), type IV collagen (**e**) and α-SMA (**f**) were measured by the qRT-PCR. The data are expressed as means ± the standard deviation of at least three different experiments. ^**^p < 0.01 versus the control; ^†^*p* < 0.05, ^††^*p* < 0.01, ^†††^*p* < 0.001 versus TGF-β1 induction

To test the effect of SFN on cell viability, MRC-5 cells were incubated with various concentrations of SFN (0, 10, 20, and 40 μM) for 24, 48, or 72 h. At a concentration of 20 μM SFN, the cell viability was 85% of the control values. In our present study, treatment with ≥40 μM SFN resulted in a significant reduction over 20% in the viability of MRC-5 cells (Fig. 2a). TGF-β1 stimulation induced proliferation of MRC-5 cells significantly and SFN treatment showed anti-proliferative effects in TGF-β1-induced proliferation (Additional file 1). TGF-β1 stimulation significantly increased fibronectin, type I collagen, type IV collagen, and α-SMA protein expression (*p* < 0.05), which was inhibited by SFN treatment (Fig. 2b). Fibronectin, type I collagen, type IV collagen, and α-SMA mRNA expression levels in MRC-5 cells were also measured by qRT-PCR (Fig. 2c, d, e, f). The expression of fibronectin and α-SMA in the TGF-β1-stimulated cells was significantly higher than that in the control cells (*p* < 0.05). SFN treatment significantly inhibited TGF-β1-induced fibronectin and α-SMA mRNA expression in fibroblasts. Western blotting and qRT-PCR showed similar results: SFN treatment attenuated TGF-β1-induced fibronectin, type I collagen, type IV collagen and α-SMA expression in fibroblasts.

### The effects of SFN on the TGF-β/SMAD signalling pathway

**Fig. 3:**
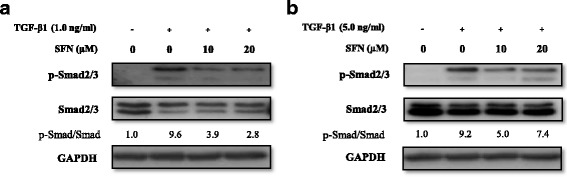
The effect of SFN on TGF-β/SMAD signalling in A549 epithelial cells and MRC-5 fibroblast cells. The cells were pre-treated for 1 h with the indicated concentrations of SFN and for 24 h with TGF-β1 (1.0 or 5.0 ng/ml). Western blotting for phosphorylated and total form of SMAD 2/3 was performed using the cell lysates of A549 epithelial cells (**a**) and MRC-5 cells (**b**). The data are expressed as means ± standard deviation of at least three different experiments

We characterised the effects of SNF treatment on TGF-β1-induced SMAD2/3 phosphorylation in an alveolar epithelia cell line and a fibroblast cell line. As shown in Fig. 3, expression of SMAD2/3 phosphorylation increased after TGF-β1 treatment, and SFN significantly reduced the TGF-β1-induced phosphorylation of SMAD2/3 protein expression in epithelial and fibroblast cells. These results suggested that the inhibition of the TGF-β-1/SMAD signalling pathway was involved in the TGF-β1-induced EMT and fibrosis. The inhibition of SFN in TGF-β1-induced phosphorylation of SMAD2/3 started in early time point within 1 h and maintained to 24 h persistently (Additional file 2).

### The effects of SFN on BLM-induced pulmonary fibrosis in an in vivo model

BLM instillation into mouse lungs induced significant pulmonary fibrosis. H&E and M-T staining of lung specimens showed that BLM instillation induced severe distortions of lung structure and accumulation of collagen fibres in the lungs. Furthermore, the expression of fibronectin and hydroxyproline significantly increased in the BLM group compared with the control group.

**Fig. 4:**
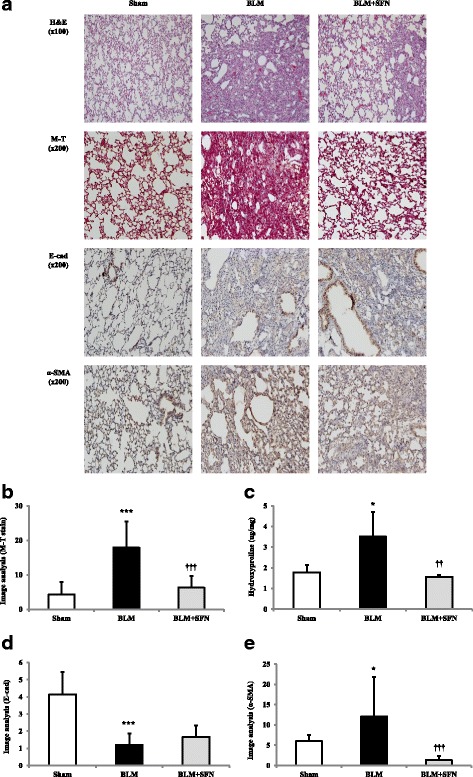
The effect of SFN on bleomycin (BLM)-induced pulmonary fibrosis. The histological results of haematoxylin and eosin, Masson’s trichrome (M-T) staining, immunohistochemistry staining of E-cad and α-SMA in lung sections (**a**). BLM induced extensive pulmonary inflammation and fibrosis. SFN treatment attenuated BLM-induced pulmonary fibrosis. M-T scores of lung histology (**b**). The M-T score increased significantly in the BLM group, and SFN treatment decreased the M-T score compared with the BLM group. The hydroxyproline assay of lung tissues (**c**). Collagen contents were evaluated by the hydroxyproline assay. The results were similar to the M-T scores. Image analysis of immunohistochemistry for E-cad (**d**) and α-SMA (**e**). SFN treatment restored E-cad expression and decreased the expression of α-SMA. The data are expressed as means ± standard deviation, *n* = 4 in each group. ^*^p < 0.05, ^***^p < 0.001 versus the control; ^††^*p* < 0.01, ^†††^*p* < 0.001 versus the BLM group

Histopathological results showed that the BLM + SFN group showed significantly attenuated BLM-induced fibrotic lesions and collagen accumulation in the lungs of mice (Fig. 4a). To confirm the effects of SFN on the histopathological changes during BLM-induced pulmonary fibrosis, the overall grade of the fibrotic changes in the lungs was scored by imaging analyses of M-T staining (M-T score). The scores of the BLM + SFN group were significantly lower than those of the BLM group (Fig. 4b).

**Fig. 5:**
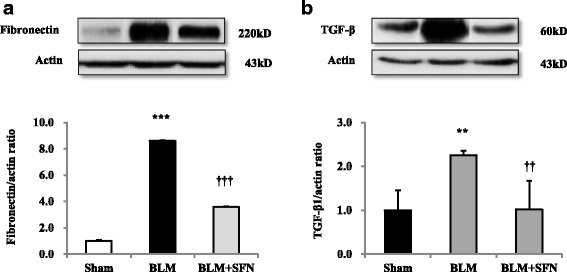
The effect of SFN on TGF-β, fibronectin expression in BLM-induced pulmonary fibrosis. The protein levels of fibronectin (**a**) and TGF-β (**b**) were measured by western blot analyses. Each experiment was performed in triplicate and repeated three times. The data are expressed as means ± standard deviation, *n* = 4 in each group. ^**^p < 0.01, ^***^p < 0.001 versus the control; ^††^*p* < 0.01, ^†††^*p* < 0.001 versus the BLM group

The collagen content in lung tissues was assessed by measuring the hydroxyproline content. Compared with the BLM group, the hydroxyproline content decreased in the BLM + SFN group (Fig. 4c). Because they are important components in pulmonary fibrosis, fibronectin and TGF-β1 were assessed by western blotting. BLM significantly increased fibronectin and TGF-β1 protein expression in lung tissues, which was attenuated by SFN treatment (Fig. 5a, b).

Immunohistochemistry was used to characterise epithelial (E-cad) and mesenchymal markers (α-SMA) from lung samples of mice treated with distilled water (control), BLM, and BLM + SFN (Fig. 4a). As expected, the control group contained many stained E-cad-positive cells on the surface of the bronchus and the alveolar wall epithelial layer. In contrast, there were decreased E-cad-positive cells in the BLM group, and more E-cad-positive cells were found in the BLM + SFN group than the BLM group (Fig. 4d). The α-SMA expression was mostly found in interstitial fibrotic areas and sporadically in epithelial tissue. The expression of α-SMA was also significantly decreased in the BLM + SFN group compared with the BLM group (Fig. 4e).

## Discussion

SFN is a natural isothiocyanate found in cruciferous vegetables with reported anti-inflammatory, bactericidal, anti-helminthic, and anti-fibrotic properties, and has been tested as an anti-tumour agent [16, 17]. Several studies have examined the efficacy of SFN in the treatment of fibrosis. In IPF fibroblasts, Artaud-Macari et al. [24] reported that SFN decreased oxidants and induced the production of Nrf2 and antioxidants, as well as the dedifferentiation of myofibroblasts. Oh et al. [22] reported that SFN inhibited the development and progression of early-stage hepatic fibrosis induced by bile duct ligation in mice. In that study, SFN was also reported to have an anti-fibrotic effect during hepatic fibrosis involving Nrf2-mediated inhibition of TGF-β/SMAD signalling and subsequent suppression of hepatic stellate cell activation and fibrogenic gene expression [22]. Under hyperglycaemic and oxidative conditions, SFN treatment prevented nephropathy, diabetes-induced fibrosis, and vascular complications [26]. Several studies have concluded that the anti-fibrotic effect of SFN involves the suppression of oxidative stress [24, 25, 27, 28]. In the present study, the anti-fibrotic effect of SFN on TGF-β1-stimulated MRC-5 cells, A549 cells, and on BLM-induced pulmonary fibrosis was found to involve the inhibition of EMT. In a previous study, SFN was reported to have inhibitory activity during oncogenic EMT [20, 21]. Shan et al. [18] reported that SFN inhibited the EMT process involving E-cad induction via the transcriptional repressors ZEB1 and Snail in bladder cancer cells.

In the present study, SFN inhibited BLM-induced pulmonary fibrosis in mice. BLM causes alveolar cell damage and pulmonary inflammation, and has been used to produce experimental pulmonary fibrosis models in different animals [29]. In the present study, BLM (5 units on days 1 and 14) was introduced intratracheally into mouse lungs to induce pulmonary fibrosis because repetitive intratracheal administration of BLM more effectively induces the chronic pulmonary fibrosis [30]. We demonstrated fibronectin overexpression and collagen overproduction after BLM treatment and found that SFN attenuated these upregulations in the mouse model. The histological changes observed by H&E and M-T staining after BLM stimulation showed massive inflammation, fibrosis, and structural distortion. In the present study, BLM was introduced into lungs, and the animals were then treated with SFN three times a week for 4 weeks. This treatment inhibited pulmonary fibrosis progression. BLM was observed to induce strong M-T staining versus the controls, and SFN significantly attenuated the severity of this staining.

Moreover, we observed that SFN inhibited BLM-induced TGF-β1 protein expression. TGF-β is a potent profibrogenic mediator and has been reported to be expressionally upregulated in many fibrotic diseases, including pulmonary fibrosis [31]. Thus, reports on the effects of TGF-β on fibrogenesis during pulmonary fibrosis have suggested that the disruption of TGF-β production or blocking of TGF-β signalling be considered as therapeutic targets of IPF [3]. TGF-β acts on multiple cell types during pulmonary fibrosis, for example, to induce EMT in alveolar or airway epithelial cells, and proliferation and differentiation into myofibroblasts in fibroblasts [3, 4, 6, 8]. TGF-β-induced EMT is an important component of the mechanisms of fibrotic pulmonary diseases [5–8]. In the present study, we observed that SFN inhibited TGF-β1-induced EMT in alveolar epithelial cells. Furthermore, immunohistochemical analysis showed that BLM downregulated E-cad and upregulated α-SMA, which are markers of EMT. In addition, SFN prevented BLM-induced changes in the expression of EMT markers in the lungs of mice. SFN also inhibited the proliferation of MRC-5 cells (a pulmonary fibroblast cell line) and suppressed the TGF-β1-induced overexpression of fibrogenic proteins (fibronectin, collagen I, and α-SMA) in these cells. The results of the mRNA assays also showed that SFN decreased the levels of TGF-β1-induced fibronectin, collagen I, and α-SMA mRNA in MRC-5 cells. In A549 alveolar epithelial cells, western blotting showed that SFN treatment resulted in TGF-β1-induced EMT, reduced E-cad expression, and increased expression of N-cad, vimentin, and α-SMA. We also observed that SFN treatment reduced the mRNA levels of TGF-β1-induced EMT-related transcriptional factors (Slug, Snail, and Twist) in A549 cells. Moreover, SFN suppressed the TGF-β1-induced phosphorylation of SMAD2/3 in MRC-5 and A549 cells, and showed a significant anti-fibrotic effect. These results suggest that the anti-fibrotic activity of SFN in pulmonary fibrosis involves the inhibition of TGF-β signalling by SMAD2/3.

In pulmonary fibroblasts, fibrous proteins, such as fibronectin, collagen I, and α-SMA, were induced by TGF-β1, and SFN treatment inhibited TGF-β1-induced fibrous protein expression. Fibronectin is a glycoprotein of the extracellular matrix and has major roles in cell adhesion, growth, migration, and differentiation, and in wound healing [32]. Altered fibronectin expression has been associated with the pathogenesis of various conditions, including cancer and fibrosis [3, 32]. Collagen is another major fibrous protein of the extracellular matrix, and its excessive deposition contributes to the pathogenesis of pulmonary fibrosis [3, 32]. In the present study, we evaluated α-SMA (a commonly used marker of myofibroblast formation), fibronectin, and collagen I levels in TGF-β1-stimulated fibroblasts in the presence or absence of SFN.

The intracellular transcriptional pathway of TGF-β including SMAD and non-SMAD pathways is well established [31]. When TGF-β receptors are activated, they undergo conformational changes that allow direct binding with SMADs and their phosphorylated products. This results in the accumulation of SMADs in the nucleus to regulate target gene transcription. When we investigated how SFN treatment affected the TGF-β1/SMAD2/3 signalling pathway, we found that it inhibited the TGF-β1-induced phosphorylation of SMAD2/3. Interestingly, Yan et al. [25] reported recently that the anti-fibrotic efficacy of SFN in BLM-induced pulmonary fibrosis involved amelioration of oxidative stress. Thus, we hypothesise that the major mechanism underlying the action of SFN probably involves inhibition of the TGF-β1-induced SMAD2/3 signalling pathway and the subsequent suppression of EMT. In the studies about antifibrotic efficacy of SFN, the dose of SFN is variable according to the route of administration such as subcutaneous injection, intraperitoneal injection, or gavage (0.5–25.0 mg/kg) [22, 25, 26]. In this study, we used SFN with minimal dose (50 μg/kg) and demonstrated antifibrotic effect in pulmonary fibrosis mice model.

## Conclusion

In summary, we found that SFN attenuated TGF-β1-induced fibrosis in MRC-5 cells, TGF-β1-induced EMT in A549 cells, and BLM-induced pulmonary fibrosis in a mouse model. These findings indicate that the major mechanism responsible for the effects of SFN involves inhibition of the TGF-β1-induced SMAD2/3 signalling pathway and subsequent EMT suppression. Based on these results, we suggest that SFN may be considered a potential treatment for IPF. Future studies should determine the optimal dosage of SFN and identify other mechanisms that contribute to pulmonary fibrosis.

## Additional files

Additional file 1: The effect of SFN in cell viability of TGF-β1-stimulated cells for 24, 48, and 72 h. In A549 cells, TGF-β1 did not induce significant proliferation (A). TGF-β1-induced proliferation of MRC-5 cells showed inhibition by treatment of SFN for 1 h (B). (PDF 315 kb)

Additional file 2: Time response in the phosphorylation of SMAD2/3 by TGF-β1 and SFN treatment. In Western blotting of both A549 cells (A) and MRC-5 cells (B) showed phosphorylation of SMAD2/3 in early time within 1 h by TGF-β1. (PDF 113 kb)

## Abbreviations

BLM: Bleomycin
COL1A1: Type I collagen
COL4A1: Type IV collagen
E-cad: E-cadherin
EMT: Epithelial-to-mesenchymal transition
FN: Fibronectin
IPF: Idiopathic pulmonary fibrosis
N-cad: N-cadherin
Nrf2: Nuclear factor erythroid-derived 2-related factor 2
qRT-PCR: Quantitative real-time polymerase chain reaction
SFN: Sulphoraphane
TGF-β: Transforming growth factor-β
α-SMA: α-smooth muscle actin
