# Supplementary figures and images for: Sulforaphane attenuates pulmonary fibrosis by inhibiting the epithelial-mesenchymal transition
Source: BMC Pharmacol Toxicol. 2018 Apr 2;19:13. doi: 10.1186/s40360-018-0204-7 (PMC5879815; doi:10.1186/s40360-018-0204-7)

**A**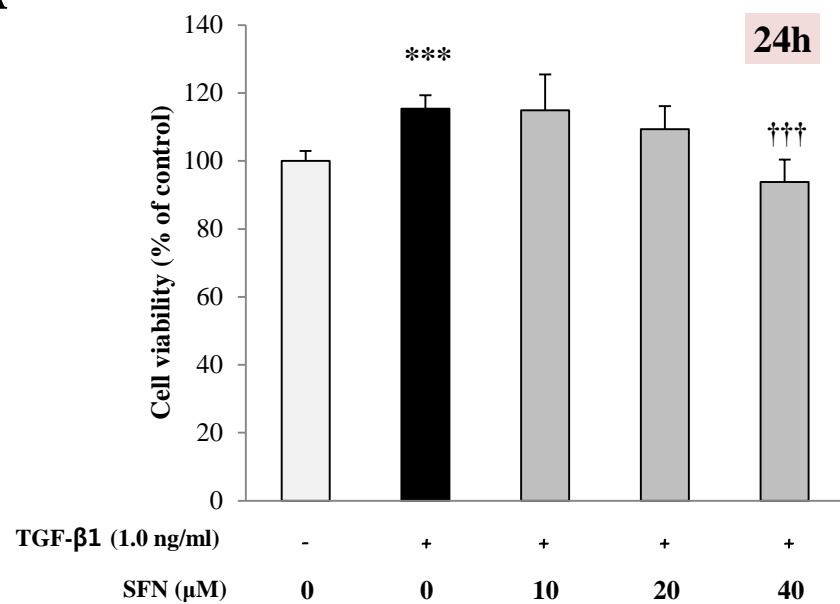**B**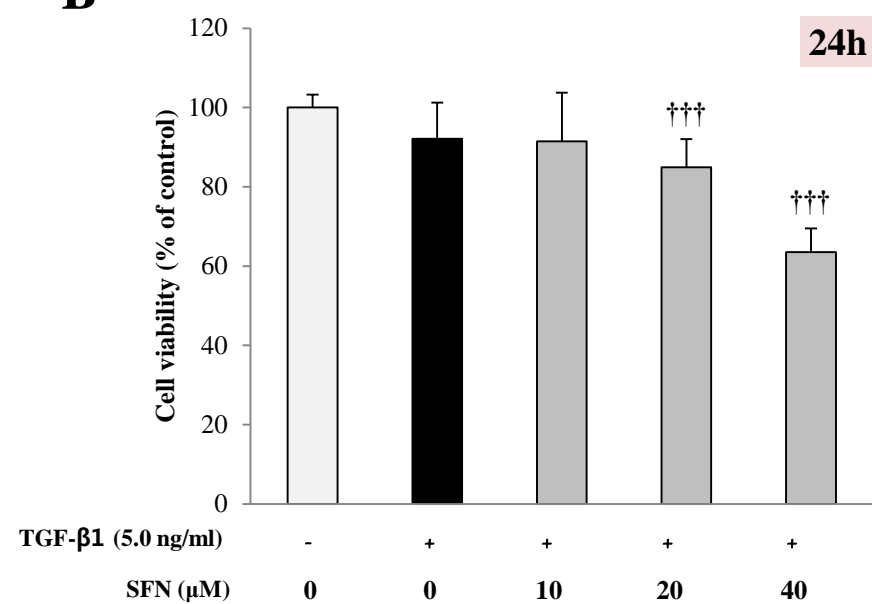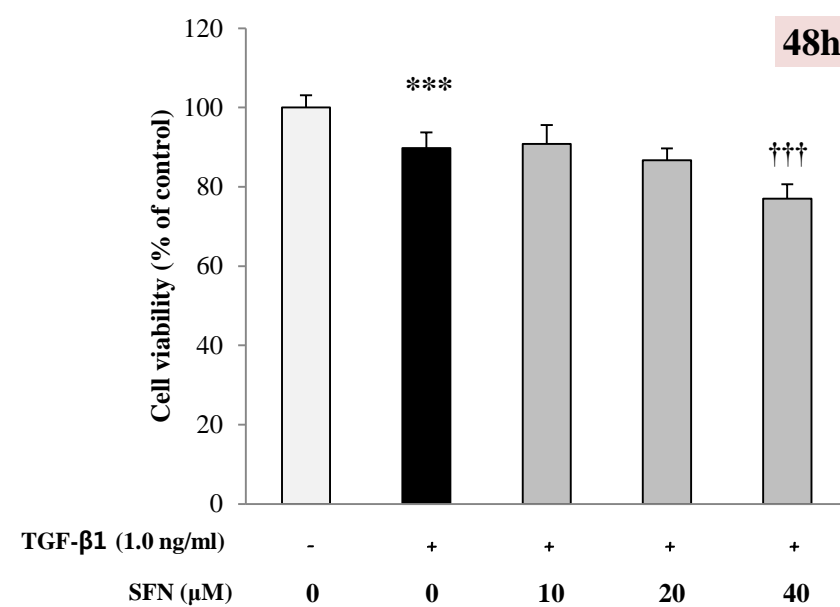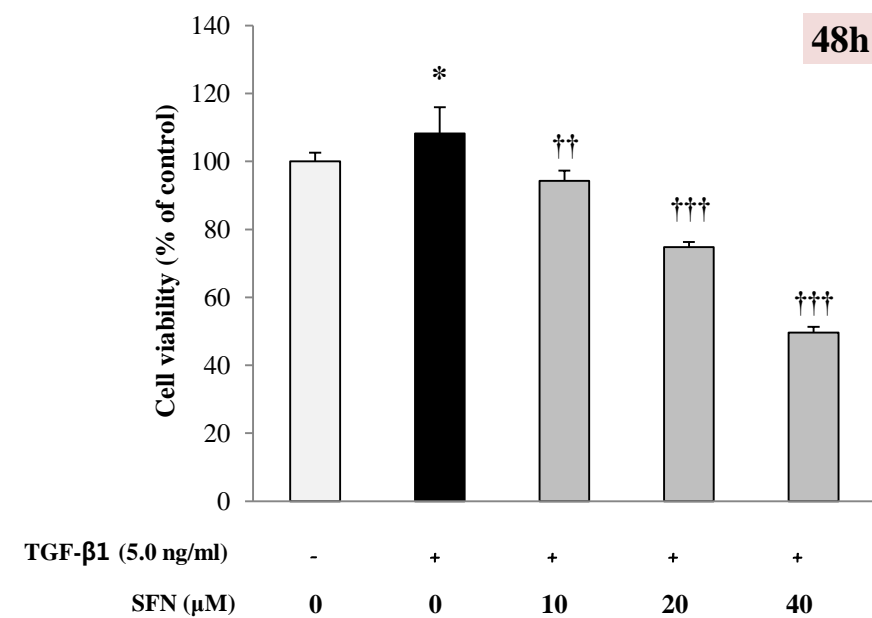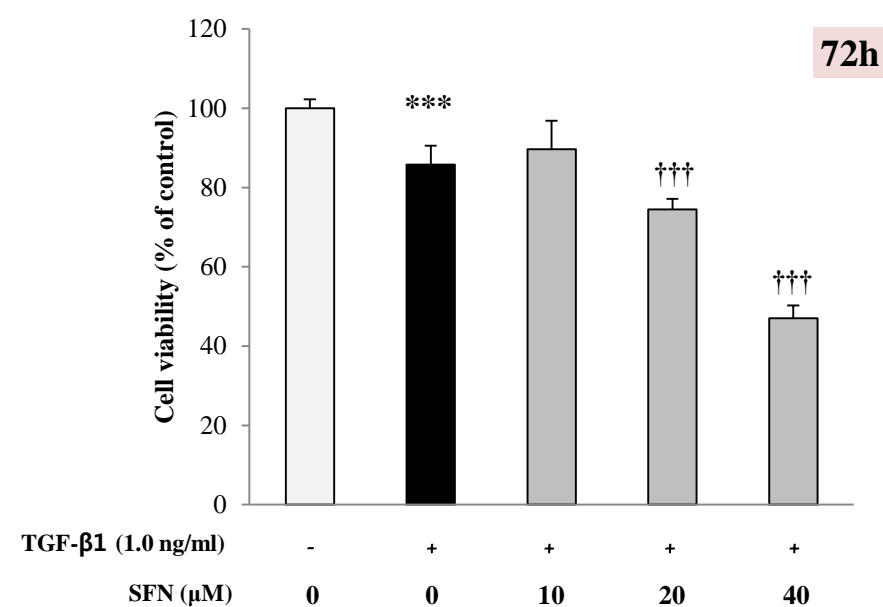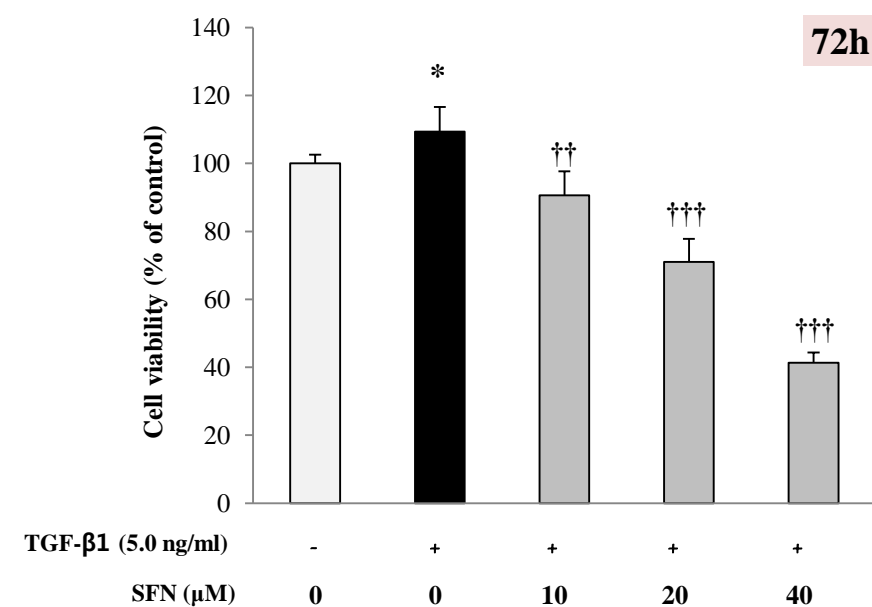

Supplement: Supplementary file 1 — The effect of SFN in cell viability of TGF-β1-stimulated cells for 24, 48, and 72 h. In A549 cells, TGF-β1 did not induce significant proliferation (A). TGF-β1-induced proliferation of MRC-5 cells showed inhibition by treatment of SFN for 1 h (B). (PDF 315 kb) [file 40360_2018_204_MOESM1_ESM.pdf]

**A**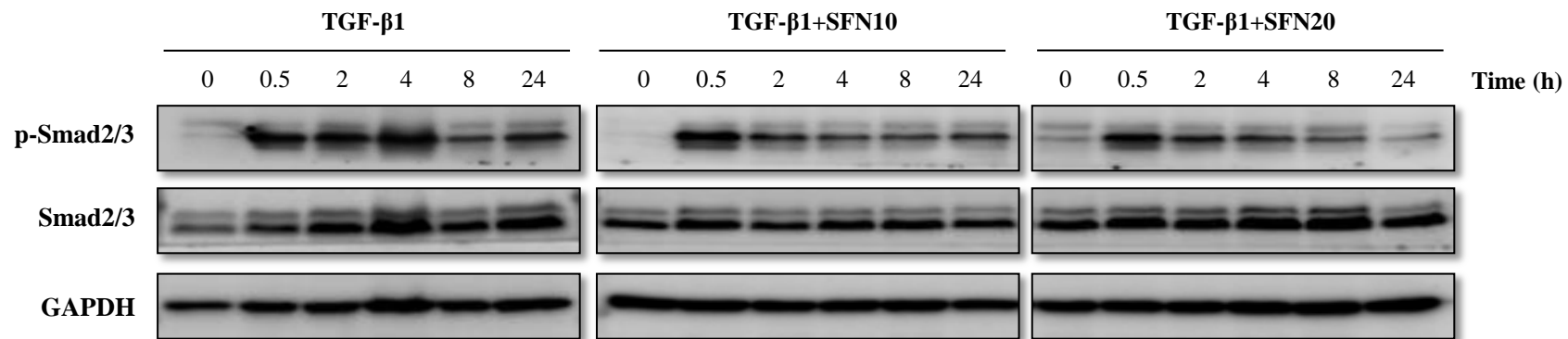**B**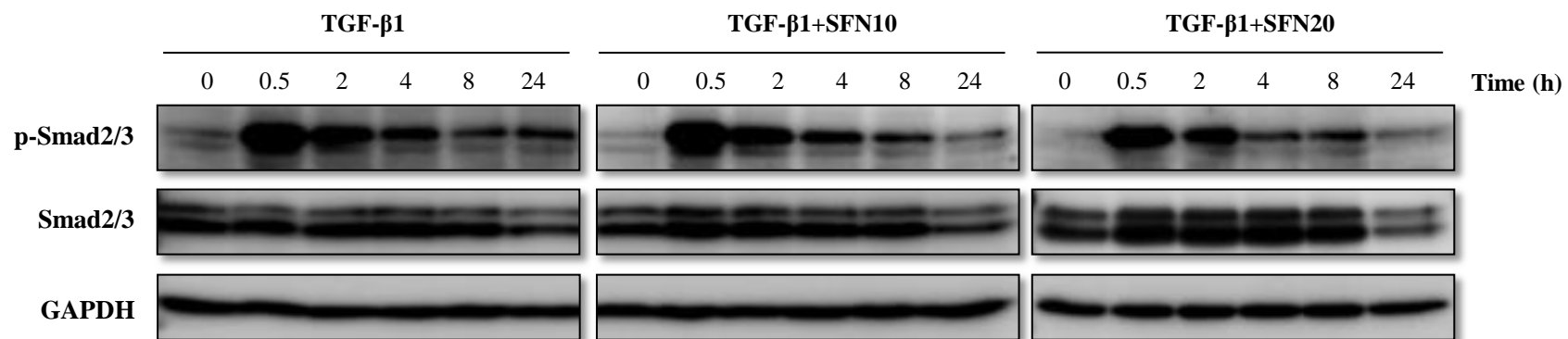

Supplement: Supplementary file 2 — Time response in the phosphorylation of SMAD2/3 by TGF-β1 and SFN treatment. In Western blotting of both A549 cells (A) and MRC-5 cells (B) showed phosphorylation of SMAD2/3 in early time within 1 h by TGF-β1. (PDF 113 kb) [file 40360_2018_204_MOESM2_ESM.pdf]
